# P2X4 signalling contributes to hyperactivity but not pain sensitization comorbidity in a mouse model of attention deficit/hyperactivity disorder

**DOI:** 10.3389/fphar.2023.1288994

**Published:** 2024-01-04

**Authors:** Sarah Bou Sader Nehme, Sandra Sanchez-Sarasua, Ramy Adel, Marie Tuifua, Awatef Ali, Amina E. Essawy, Sherine Abdel Salam, Walid Hleihel, Eric Boué-Grabot, Marc Landry

**Affiliations:** ^1^ University of Bordeaux, CNRS, Institute of Neurodegenerative Diseases, IMN, UMR 5293, Bordeaux, France; ^2^ Department of Biology, Faculty of Arts and Sciences, Holy Spirit University of Kaslik, Jounieh, Lebanon; ^3^ Faculty of Health Sciences, University of Jaume I, Castellon, Spain; ^4^ Zoology Department, Faculty of Science, Alexandria University, Alexandria, Egypt

**Keywords:** attention deficit/hyperactivity disorders, pain sensitization, purines, P2X4, anterior cingulate cortex, posterior insula

## Abstract

**Introduction:** Attention deficit/hyperactivity disorder (ADHD) is a common neurodevelopmental disorder characterized by hyperactivity, inattention, and impulsivity that often persist until adulthood. Frequent comorbid disorders accompany ADHD and two thirds of children diagnosed with ADHD also suffer from behavioural disorders and from alteration of sensory processing. We recently characterized the comorbidity between ADHD-like symptoms and pain sensitisation in a pharmacological mouse model of ADHD, and we demonstrated the implication of the anterior cingulate cortex and posterior insula. However, few studies have explored the causal mechanisms underlying the interactions between ADHD and pain. The implication of inflammatory mechanisms has been suggested but the signalling pathways involved have not been explored.

**Methods:** We investigated the roles of purinergic signalling, at the crossroad of pain and neuroinflammatory pathways, by using a transgenic mouse line that carries a total deletion of the P2X4 receptor.

**Results:** We demonstrated that P2X4 deletion prevents hyperactivity in the mouse model of ADHD. In contrast, the absence of P2X4 lowered thermal pain thresholds in sham conditions and did not affect pain sensitization in ADHD-like conditions. We further analysed microglia reactivity and the expression of inflammatory markers in wild type and P2X4KO mice. Our results revealed that P2X4 deletion limits microglia reactivity but at the same time exerts proinflammatory effects in the anterior cingulate cortex and posterior insula.

**Conclusion:** This dual role of P2X4 could be responsible for the differential effects noted on ADHD-like symptoms and pain sensitization and calls for further studies to investigate the therapeutic benefit of targeting the P2X4 receptor in ADHD patients.

## Introduction

Attention deficit and hyperactivity disorder (ADHD) is classified in the DSM-IV (Diagnostic and Statistical Manual of mental Disorders) (American Psychiatric Association, 2013) as a heterogeneous neurodevelopmental disorder manifested by varying levels of hyperactivity, impulsivity, and inattention in humans. ADHD is the most common neuropsychiatric disorder with a prevalence of 8%–12% in children (Danielson et al., 2018) that remains steady over the last decades (Polanczyk et al., 2014; Song et al., 2021). ADHD exists as three sub-types: Inattentive, hyperactive-impulsive, or combined, and the T.O.V.A test can discriminate between these sub-types (Greenberg et al., 1991). ADHD continues into adulthood in up to 50% of the patients diagnosed during childhood (Faraone et al., 2006), and up to 80% for patients of the combined subtype of ADHD (van Lieshout et al., 2016). Adult symptoms are variable and may include functional impairments (Simon et al., 2009). The Conners’ Adult ADHD Rating Scales (CAARS) is used to determine whether an individual may have ADHD and what is the symptoms severity.

In clinics, ADHD patients report impaired perceptual functions (Fuermaier et al., 2018; Panagiotidi et al., 2018; Dellapiazza et al., 2021) and pain sensitivity (Treister et al., 2015). ADHD patients have increased risks of pain disorders and high prevalence of pain among adults with ADHD can reach up to 80% (Lensing et al., 2013; Stray et al., 2013). ADHD severity positively predicts pain sensitivity in human adolescent males, and it is associated to larger physiological response as measured by skin conductance level (Northover et al., 2015). Accordingly, attentional processes have been shown to regulate pain transmission through the modulation of brain networks (Kucyi et al., 2013) and descending pathways (Villemure and Bushnell, 2002; Sprenger et al., 2012). Reciprocally, chronic pain increases impulsivity (Ko et al., 2013) and induces attentional and cognitive deficits in human patients (Moore et al., 2019; Kasahara et al., 2020) and preclinical animal models (Higgins et al., 2015). Among 153 patients with chronic pain, 72.5% had a CAARS and frequently experience symptoms such as hyperactive behavior and other clinical characteristics of ADHD (Kasahara et al., 2020).

The understanding of the mechanisms underpinning ADHD and pain comorbidity requires to investigate animal models of ADHD with good face, construct and predictive validity. Because alterations of dopamine transmission are considered as significant factors for the progression of the disease (Albrecht et al., 2015), a mouse model of ADHD by lesioning dopaminergic fibers with neonatal intra-cerebroventricular injection of 6-hydroxydopamine (6-OHDA) was developed and validated (Sontag et al., 2010; Bouchatta et al., 2018; 2020). In addition to hyperactivity, inattention and impulsivity, the 6-OHDA mice exhibited a marked decrease of withdrawal thresholds to thermal and mechanical stimuli, suggesting that ADHD-like conditions increase nociceptive sensitivity (Bouchatta et al., 2022; Meseguer-Beltrán et al., 2023; Sifeddine et al., 2023). These previous studies indicated that ADHD and pain sensitization are mutually worsening comorbid disorders. They also pointed to a key role of the anterior cingulate cortex (ACC) hyperactivity that alters the ‘ACC–posterior insula (PI)’ circuit, and triggers pain sensitization (Bouchatta et al., 2022).

ADHD patients display a large heterogeneity in symptoms and cognitive performances (Castellanos and Tannock, 2002; Nigg et al., 2005). The exact origin of this heterogeneity remains unknown. However, it has been suggested that inconsistent neurotransmission in neural circuits that engage the prefrontal cortex may account for fluctuations in attentional processing and cognitive performance and hence, for intra-individual variability (Russell et al., 2006). Neurotransmission impairments can be caused by alterations in neurotransmitter release or binding (Posner et al., 2020), neuroglia interactions (Oades et al., 2010; Zhang et al., 2022), or neuroinflammatory processes (Leffa et al., 2018; Corona, 2020).

Purinergic signalling influences numerous physiological processes including neuromodulation, synaptic plasticity, neuroinflammation and neuron-glia communication and is also involved in major CNS disorders. ATP is released by neurons as well as by astrocytes or microglia and activates different P2 receptors and adenosine receptors after extracellular ATP catabolism by ectonucleaotidases expressed in various cell types (Rodrigues et al., 2015). In many pathological conditions, the increase of ATP release exacerbates ATP signalling promoting astrogliosis, activation of microglia and inflammatory responses (Rodrigues et al., 2015). Among ATP P2 receptors, P2X receptors are trimeric ATP-gated cation channels made from 7 different subunits (P2X1–P2X7) encoded by 7 genes in mammals. P2X4 is the main subunit in neurons and microglia, and possibly astrocytes (Suurväli et al., 2017). It is the most widely distributed P2X receptor in the brain and is likely to modulate cortical circuits that underpin attentional and cognitive processes as well as pain perception. Since P2X4 is involved in various neurological and psychiatric disorders (Lindberg et al., 2015; Duveau et al., 2020; Montilla et al., 2020), we made the hypothesis that P2X4 could play an important role in ADHD symptoms and comorbid pain by modulating microglia activation and neuronal transmission in the ACC and/or the PI.

To address this question, we generated an ADHD-like model by neonatal injection of 6-OHDA in a mouse line carrying a global deletion of the P2X4 receptor (P2X4KO) (Sim et al., 2006). To identify possible neural pathways that are influenced by P2X4 signalling in the ADHD model, we explored the expression of neuroinflammatory and microglial activation markers in the ACC and PI of 6-OHDA wild-type (WT) and P2X4KO mice. We further measured hyperactivity, the most common symptom of ADHD, and assessed pain sensitisation.

## Materials and methods

**FIGURE 1:**
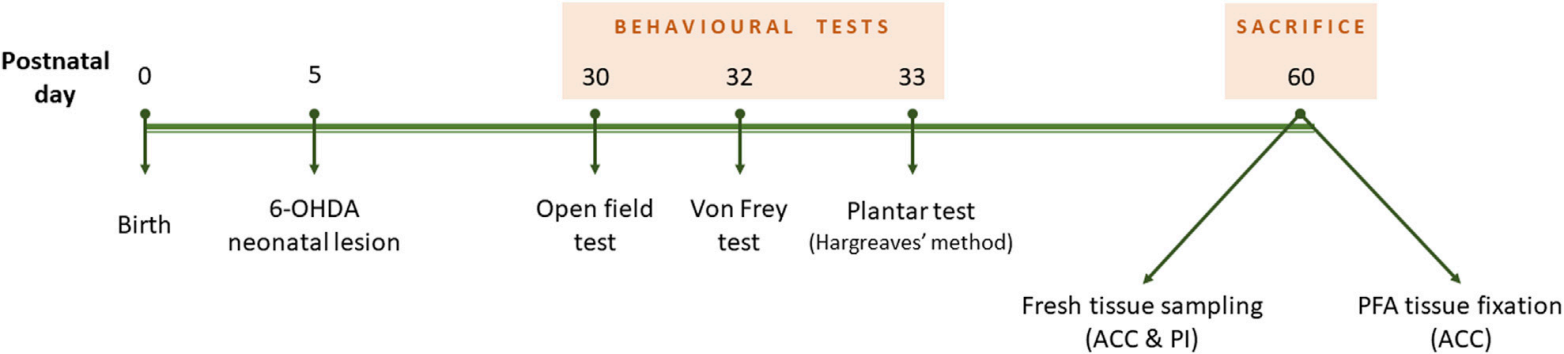
Experiment design timeline. Neonatal 6-OHDA injection was performed at postnatal day 5. Behavioural tests lasted from P30 to P33, and mice were culled at 2 months old.

The time course of the experiments is described in Figure 1.

### Animals

C57BL/6 (Janvier Labs, France) and P2X4KO (EOPS facility, Bordeaux Neurocampus) pregnant females and their newborn pups were housed under a 12 h light/dark cycle (lights on at 7 a.m.), provided with water and food *ad libitum*, at the animal facility of the University of Bordeaux. The study was approved by the local ethical committee and the French Ministry of Higher Education, Research and Innovation (authorization APAFIS#27681 and APAFIS#21135). Procedures were conducted in conformity with the approved institutional protocols. Efforts were made to reduce animal suffering.

### 6-OHDA neonatal injection (P5 surgery)

At postnatal day 5 (P5), male and female pups were submitted to hypothermal anesthesia and received an intracerebroventricular injection (AP −2 mm, ML ±0.6 mm, DV−1.3 mm from Bregma) of 25 μg 6-OHDA hydrobromide (Sigma-Aldrich, France) dissolved in 3 μL 0.1% ascorbic acid, or vehicle, following a published protocol (Bouchatta et al., 2018). A pretreatment with desipramine hydrochloride (20 mg/kg s.c., Sigma-Aldrich, France) was performed 30 min before surgery to prevent the depletion of noradrenergic neurons. After surgery, the pups were placed back with the mother, kept under constant observation, and provided with enriched food (#SAFED113, Safe-lab, Augy, France) to improve recovery. After weaning, mice were housed in cages of two to five animals to reduce stress due to isolation.

### Behavioural procedures

For all the behavioural paradigms, mice were habituated to the researcher for 4 days before starting the procedures and to the testing room 30 min before performing each test. Behavioural procedures started at P30. The tests were conducted during the day, with a dim light. All apparatuses were cleaned using a 30% ethanol solution between trials and animals. All mice performed all the paradigms.

1. **Open Field:** spontaneous locomotor activity was assessed through the open field test. Mice were placed in the center of the arena (measurements: 40 cm × 40 cm × 40 cm; Ugo Basile, Gemonio, Italy) and allowed to freely explore for 10 min. Animals were recorded using a video tracking system (EthoVision XT15, Noldus, Wageningen, Netherlands). Distance traveled (cm) and speed (cm/s) were quantified.
2. **Von Frey test:** nociceptive response to mechanical stimulus was assessed using von Frey setup (Ugo Basile, Gemonio, Italy), as described previously (Bouchatta et al., 2022). During 30 min, mice were habituated to individual cages with a mesh floor. The plantar surface of the hind paws was stimulated by calibrated von Frey filaments of different grams to set the withdrawal threshold. For both hind paws, three to five measurements were registered, with an interval of 30 s between each. The grams of the filament at which the mouse withdrew its paw was considered to be the mechanical pain threshold value.
3. **Plantar test (Hargreaves’method):** nociceptive response to thermal stimulus was assessed using Plantar test setup (Ugo Basile, Gemonio, Italy), as described previously (Bouchatta et al., 2022). During 30 min, mice were habituated to individual cages with a glass pane floor. The plantar surface of the hind paws was stimulated by an infrared (IRed) generator (IRed intensity of 50, cut-off time set at 15 s) to establish the thermal pain threshold. For both hind paws, the latency to paw withdrawal was recorded, and three measurements per paw were performed with an interval of 2 min between each.

### Tissue preparation for immunofluorescence

Mice were anesthetized with ketamine (100 mg/kg i.p., Virbac, France) and xylazine (20 mg/kg, i.p., Virbac, France) and were transcardially perfused with 0.9% NaCl containing 0.01% heparin, followed by fixative (4% paraformaldehyde (PFA) in 0.1 M phosphate buffered (PB), pH 7.4). The brains were removed and immersed in 4% PFA overnight at 4°C. After post-fixation, brains were cryoprotected in 12.5% sucrose in 0.01 M phosphate-buffered saline (PBS) at 4°C for 2 days. Coronal sections (25 μm) were obtained using a cryostat (CM3050S, Leica, Heidelberg, Germany).

### Immunofluorescence staining

The sections were washed three times, for 10 min each, in PBS 0.1M. They were incubated for 1 h, at room temperature, in a blocking solution consisting of PBS BSA 1% containing 0.3% Triton X-100, and then with anti-Iba1 (rabbit, dilution 1:2000, Wako, Japan) in a solution of PBS BSA 1%, overnight at 4°C. The sections were rinsed again three times, for 10 min each, in PBS 0.1M. They were incubated with a secondary Alexa fluor 568 goat anti-rabbit antibody (dilution 1:500, Invitrogen, MA, United States), for 2 h at room temperature. A final three-times washing step was performed, and the sections were mounted on Superfrost slides (Fisher Scientific, Illkirch, France) and coverslipped with Fluoromount G mounting medium (Invitrogen).

### Image acquisition and analysis

Images of the ACC were acquired using a confocal microscope (Leica TCS SP5), with the 20x oil immersion objective. Two optical planes for each picture were chosen, and five, non-overlapping cells were selected randomly. Six to 7 mice were analyzed per group, with at least 400 cells quantified per group. The analysis was performed using FIJI and FracLac for ImageJ software as previously described (Espinosa-Fernández et al., 2019). Four morphological parameters were assessed: cell perimeter, cell area, fractal dimension, and lacunarity. Cell perimeter was quantified based on the outline of the cell shape, while cell area was measured through the total number of pixels in the filled shape of the cell image. Fractal dimension was used for the interpretation of cell complexity and the characterization of microglial forms. A higher fractal dimension implies a more branched and complex structure. Lacunarity was associated with fractal dimension to describe cell heterogeneity. It is indicative of morphological changes and those of the soma. The lowest the lacunarity, the more homogenous the cell image is (Fernández-Arjona et al., 2017; Espinosa-Fernández et al., 2019).

### Tissue preparation for RT-qPCR procedure

Mice were anesthetized with ketamine (100 mg/kg i.p., Virbac) and xylazine (20 mg/kg, i.p., Virbac) and then sacrificed by decapitation. The ACC and PI were rapidly dissected under a binocular, under sterile conditions, and frozen in dry ice. The samples were stored at −80°C to avoid RNA degradation.

### RNA extraction

RNA of ACC and PI was extracted using the chloroform/isopropanol method. Briefly, 500 μL of the TRI-Reagent (Euromedex, France) was added to each sample to homogenize and lyse the tissue. Chloroform (Sigma-Aldrich, France) was then used to separate the RNA (aqueous phase) from the DNA and proteins (organic phase). The aqueous phase was transferred to a new and clean tube, and isopropanol (Fisher Scientific) was added to precipitate the RNA. Ethanol 75% was utilized to wash the RNA pellet. The samples were then incubated with DNAse I (Fisher Scientific) to eliminate DNA residues. Finally, RNA quantification was performed by measuring the absorbance at 260 nm using a spectrophotometer, and its quality was checked by capillary electrophoresis with the bioanalyzer 2,100 (Agilent, CA, United States).

### RT-qPCR

cDNA synthesis was performed using 2ug of RNA, the Maxima Reverse Transcriptase enzyme (Fisher Scientific), and a mix of random and oligo dT primers (Fermentas, MA, United States) at 150 ng/μL and 200 ng/μL, respectively. Transcript-specific primers, 2 μL of cDNA at 2 ng/μL, and LightCycler 480 SY Green Master (Roche, Basel, Switzerland) in a final volume of 10 μL were used for real-time PCR. The list of primers is presented in Supplementary Table S4. The relative gene expression to the vehicle-injected group (sham group) was calculated by using the 2^−ΔΔCT^ method for each reaction. Two reference genes were selected for each area of interest: Gusb (glucuronidase beta) and Ubc (Ubiquitin C) for the PI, Gapdh (Glyceraldehyde-3-Phosphate Dehydrogenase) and Nono (Non-POU-domain-containing, Octamer binding protein) for the ACC.

### Statistical analyses

The statistical analyses were conducted using GraphPad software (GraphPad Prism V9 software, GraphPad, La Jolla, CA, United States). Gaussian distribution was determined using the Shapiro-Wilk test. For Iba1 staining data, the non-parametric two-tailed Unpaired Mann-Whitney U test for two-sample comparisons was performed, since the data did not meet normality. For RT-qPCR results, a one-tailed (RT-qPCR screening in the ACC data) or two-tailed (RT-qPCR screening in the PI data) Unpaired *t*-test were carried out, since the data reached normality. To analyse behavioural tests data, two-tailed Unpaired *t*-test was applied (Open Field, Plantar test and amplitude of changes in thermal threshold). Since Von Frey test results did not reach normality, the two-tailed Unpaired Mann-Whitney U test for two-sample comparisons was carried out. The amplitude of changes in mechanical threshold was also analysed by Mann-Whitney U test. All data were expressed as the mean ± standard error of the mean (SEM) and statistical tests were performed with probability set at *p* < 0.05. We also indicated the t-value (t, *t*-test), degree of freedom (df, *t*-test), U-value (U, Mann-Whitney U test).

## Results

### P2X4 receptor deletion prevents locomotor hyperactivity in ADHD-like conditions

**FIGURE 2:**
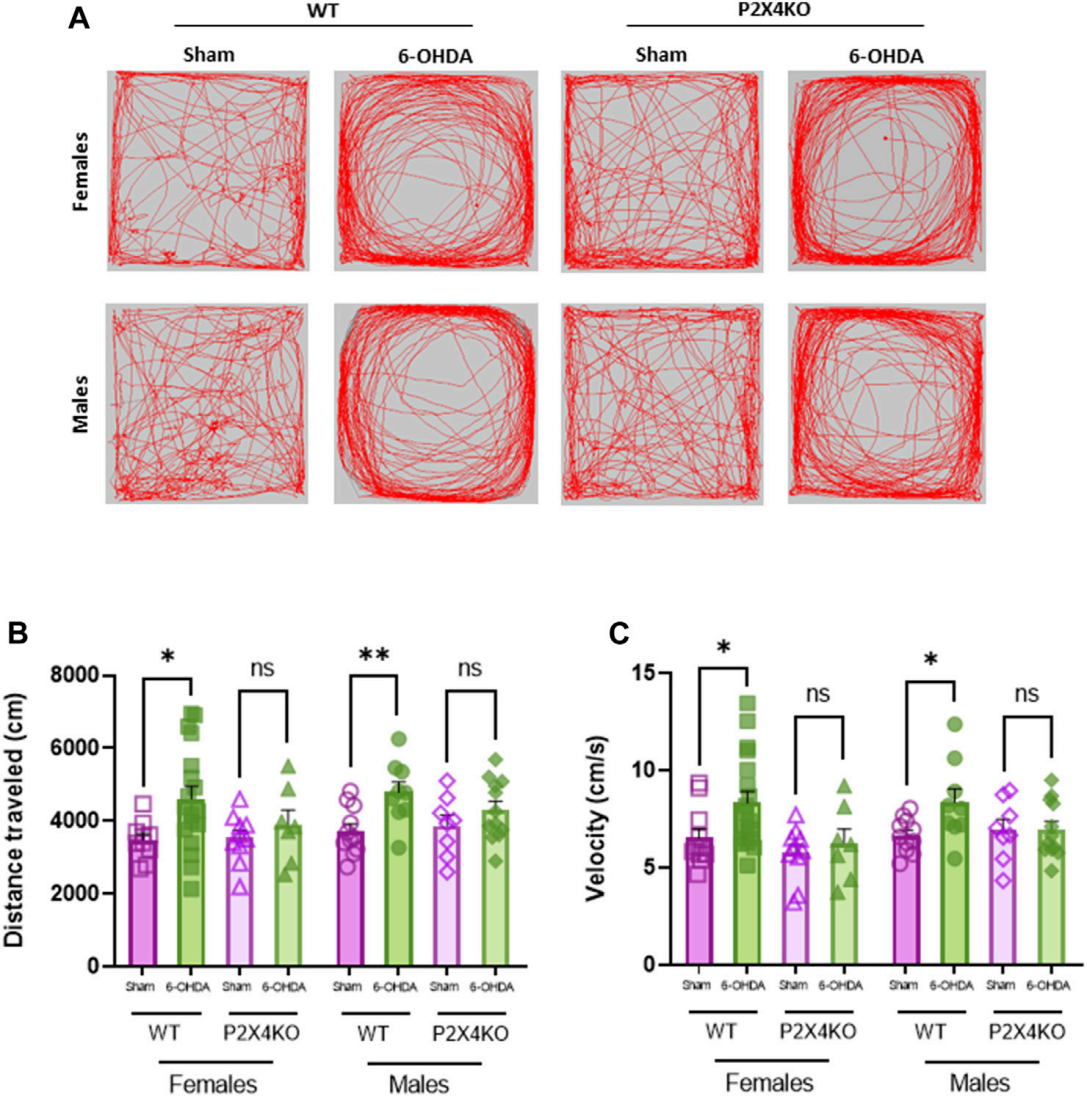
Dopaminergic lesion at P5 does not increase spontaneous locomotor activity in P2X4KO mice in both sexes. Representative images of locomotor activity traces of females (upper row) and males (bottom row) during open field test **(A)**. Distance travelled (cm) **(B)** and velocity (cm/s) **(C)** in the open field arena for female sham-WT C57BL6/J mice (empty purple squares), female 6-OHDA-WT mice (filled green squares), female sham-P2X4KO mice (empty purple triangles), female 6-OHDA-P2X4KO mice (filled green triangles), male sham-WT C57BL6/J mice (empty purple circles), male 6-OHDA-WT mice (filled green circles), male sham-P2X4KO mice (empty purple rhombi), male 6-OHDA-P2X4KO mice (filled green rhombi). Data are presented as mean ± SEM (n = 7–18 per group) and analysed with two-tailed Unpaired *t*-test (*p < 0.05; **p < 0.01; ns = not significant).

We evaluated spontaneous locomotor hyperactivity in WT and P2X4KO mice (females and males) with the open field test (Figure 2). We observed that 6-OHDA-WT animals travelled significantly more distance than sham-WT mice (Figure 2B, Supplementary Table S1A, B, two-tailed Unpaired *t*-test). Furthermore, 6-OHDA-WT group showed higher velocity than sham-WT mice (Figure 2C, Supplementary Table S1A, B).

In contrast, neonatal 6-OHDA lesion did not significantly increase the locomotor activity in P2X4KO female mice nor P2X4KO males compared with sham-P2X4KO females and males (Supplementary Table S1A, B).

We did not observe significant differences in the distance travelled nor the velocity between sham-WT and sham-P2X4KO groups. When comparing 6-OHDA groups, no significant differences were found either in distance travelled or velocity (Supplementary Table S1A, B).

We did not observe significant differences when comparing females and males of each condition in both mouse lines (WT and P2X4KO) neither in distance travelled nor in velocity (Supplementary Table S1G).

These results indicated that the lack of the P2X4 receptor prevented the development of spontaneous locomotor hyperactivity induced by neonatal 6-OHDA injection.

### P2X4 receptor deletion induces sensitization to thermal stimuli in basal but not ADHD-like conditions

Mechanical and thermal pain sensitivity were assessed with Von Frey and Plantar (Hargreaves’ method) tests, respectively (Bouchatta et al., 2022).

**FIGURE 3:**
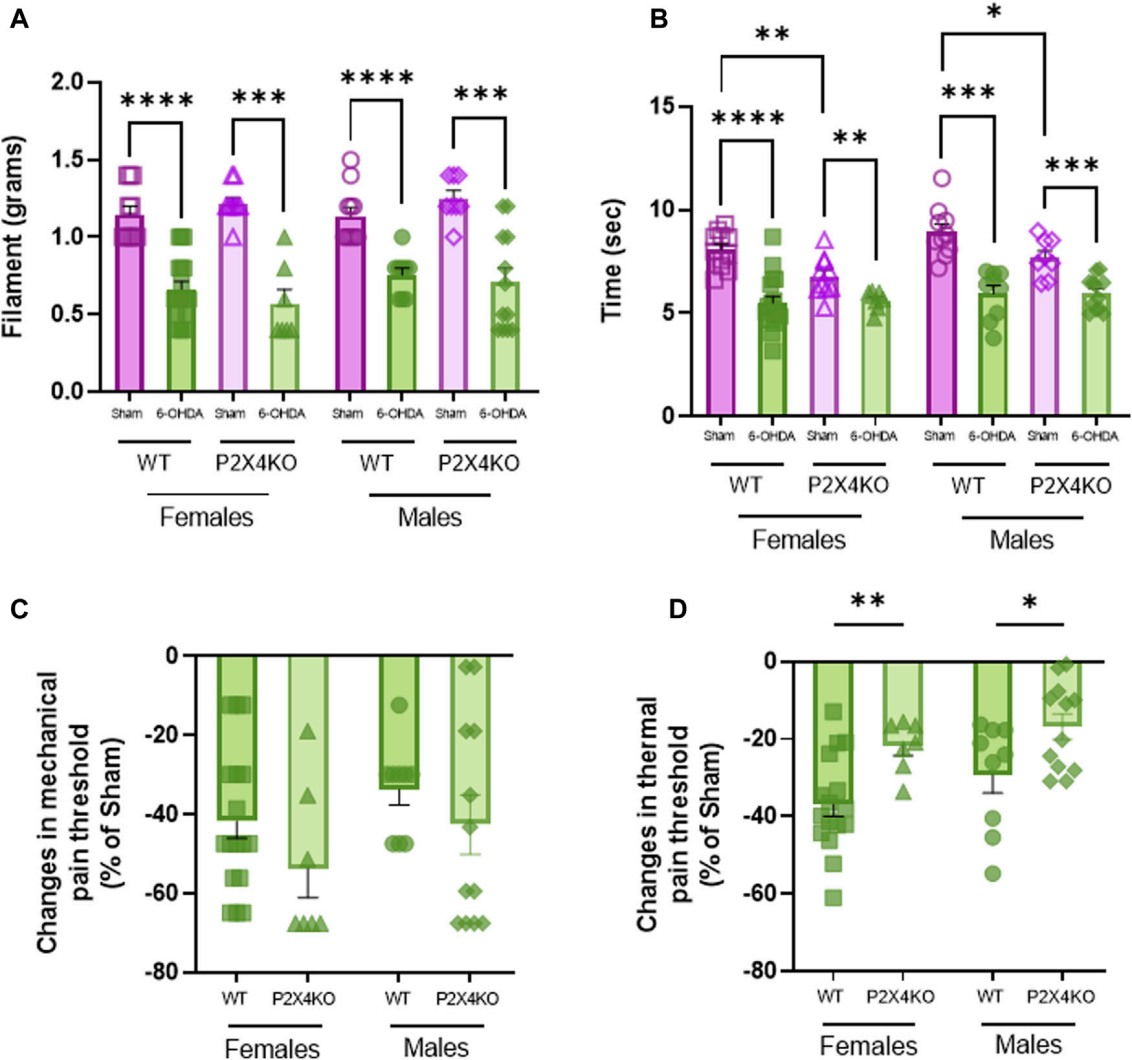
Thermal and mechanical sensitivity displayed by WT and P2X4KO mice. Mechanical threshold (grams of filament) in Von Frey test **(A)** and thermal threshold (seconds) in Plantar test (Hargreaves’ method) **(B)** of female sham-WT C57BL6/J mice (empty purple squares), female 6-OHDA-WT mice (filled green squares), female sham-P2X4KO mice (empty purple triangles), female 6-OHDA-P2X4KO mice (filled green triangles), male sham-WT C57BL6/J mice (empty purple circles), male 6-OHDA-WT mice (filled green circles), male sham-P2X4KO mice (empty purple rhombi), male 6-OHDA-P2X4KO mice (filled green rhombi). Amplitude of changes in mechanical threshold (Von Frey test) **(C)** and latency of paw withdrawal (Plantar test) **(D)** in 6-OHDA-WT and 6-OHDA-P2X4KO as compared with WT and P2X4KO sham groups in both sexes. Data presented as mean ± SEM (n = 7–17 per group). Von Frey test data and amplitude of changes in mechanical threshold were analysed with two-tailed Unpaired Mann-Whitney U test. Plantar test data and amplitude of changes in thermal threshold were analysed with two-tailed Unpaired *t*-test (*p < 0.05; **p < 0.01; ***p < 0.001; ****p < 0.0001).

In the von Frey test, WT mice that received neonatal 6-OHDA injection displayed a significant decrease in the mechanical pain threshold compared with sham animals (Figure 3A, Supplementary Table S1C, D, two-tailed Unpaired Mann-Whitney U test). Moreover, P2X4KO mice injected with 6-OHDA at P5 also showed lower mechanical pain threshold than sham-P2X4KO group (Supplementary Table S1C, D). When comparing WT and P2X4KO groups, we did not find any significative difference in shams (Supplementary Table S1C, D). Similarly, we did not observe significant differences between 6-OHDA groups (Supplementary Table S1C, D).

With the plantar test, we observed in both WT and P2X4KO groups that 6-OHDA females and males were significantly more sensitive to thermal stimulation than sham females and males (Figure 3B, Supplementary Table S1E, F, two-tailed Unpaired *t*-test). Interestingly, P2X4KO sham mice displayed significantly higher thermal pain sensitization than WT sham mice and this effect is irrespective of sex (Supplementary Table S1E, F). No significant difference was observed when comparing the 6-OHDA groups (Supplementary Table S1E, F).

We did not find significant differences between males and females from each experimental condition neither in mechanical nor thermal pain threshold (Supplementary Table S1G).

When data was expressed in percentage of changes with respect to sham groups of each mouse line (WT or P2X4KO), we observed that differences in thermal pain threshold between sham and 6-OHDA-P2X4KO mice were significantly lower than between sham and 6-OHDA-WT groups, determined by two-tailed Unpaired *t*-test for females and males (Figure 3D, Supplementary Table S1E, F). Regarding mechanical sensitivity, we did not observe significant differences in the percentage of changes between WT females and P2X4KO females, nor between WT males and P2X4KO males (Figure 3C, Supplementary Table S1C, D).

Our finding suggested that neonatal dopaminergic lesion induced mechanical and thermal pain sensitization independently of P2X4 receptor suppression, but the lack of this purinergic receptor decreased thermal pain threshold in basal conditions.

### The P2X4 receptor modulates microglia reactivity

**FIGURE 4:**
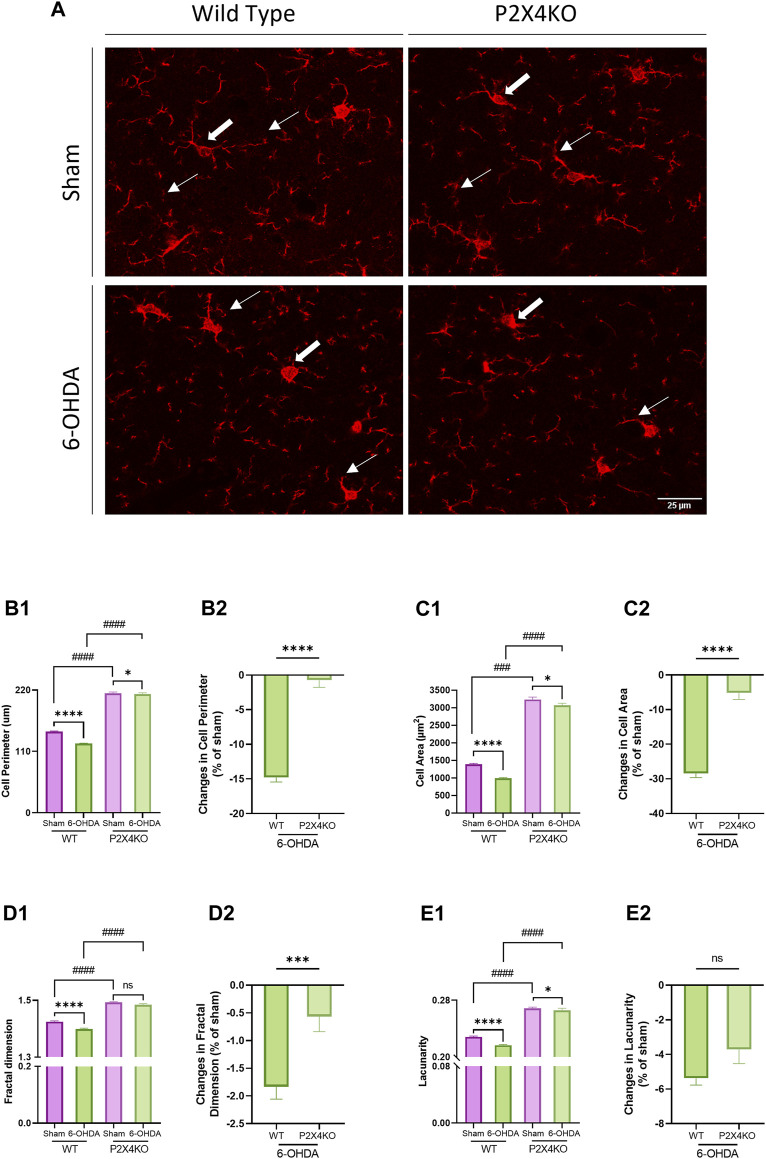
Microglia morphology in the anterior cingulate cortex (ACC). Representative images of microglia in the ACC of sham and 6-OHDA WT and P2X4KO mice **(A)**. The arrows point to microglia with a complex branched morphology (thin arrows) and a small cell soma (thick arrows) in sham mice and to a de-ramified morphology (thin arrows) with an enlarged cell soma (thick arrows) in 6-OHDA mice. Five microglial cells were quantified per picture: cell perimeter (μm) **(B1)**, cell area (μm^2^) **(C1)**, fractal dimension **(D1)**, and lacunarity **(E1)** were determined. Amplitude of changes in cell perimeter **(B2)**, cell area **(C2)**, fractal dimension **(D2)**, and lacunarity **(E2)** were assessed in 6-OHDA-WT and 6-OHDA-P2X4KO mice as compared to sham. Data are presented as mean ± SEM (n = 6–7 mice per group) and were analysed with a two-tailed Unpaired Mann-Whitney U test (*p < 0.05; ***p < 0.001; ****p < 0.0001; ####*p* < 0.0001; ns = not significant).

Microglia activation was previously described in the spontaneously hypertensive rat (Zhang et al., 2022), and the 6-OHDA mouse (Meseguer-Beltrán et al., 2023) models of ADHD. We decided to verify microglia reactivity in our conditions, and to investigate the consequences of P2X4 deletion on microglia changes. We performed Iba1 staining to analyse morphological modifications in the ACC to compare WT and P2X4KO, and to explore possible differences in microglial reactivity between the two genotypes under ADHD-like vs. sham conditions (Figure 4A). For this purpose, we used quantitative assessments of the cell perimeter and area as indices of the ameboid-like or ramified morphological shape of microglia, and the fractal dimension and lacunarity as indices of the branching complexity (Leyh et al., 2021).

In sham animals, we observed an increase in the baseline homeostatic parameters when P2X4 was deleted compared to WT mice. Cell perimeter, cell area, fractal dimension, and lacunarity exhibited significantly higher values in sham-P2X4KO mice compared to sham-WT mice (Figure 4B1–E1, Supplementary Table S2A, two-tailed Unpaired Mann-Whitney test). Similarly, ADHD-like conditions also resulted in increased cell perimeter, cell area, fractal dimension, and lacunarity in P2X4KO as compared to WT animals (Figure 4B1–E1, Supplementary Table S2B). This suggested that P2X4 receptor deletion influenced microglial morphology toward a more pronounced homeostatic phenotype.

In both genotypes, 6-OHDA neonatal injection caused changes in microglia morphology. Cell perimeter, cell area, fractal dimension, and lacunarity appeared significantly lower in 6-OHDA-WT mice as compared to the sham animals (Figure 4A1, Figure 4B1–E1, Supplementary Table S2C, two-tailed Unpaired Mann-Whitney test). In 6-OHDA-P2X4KO mice, cell perimeter, cell area, and lacunarity displayed decreased values compared to sham-P2X4KO mice, with no significant difference in fractal dimension (Figure 4A1, Figure 4B1–E1, Supplementary Table S2D). Overall, lower values for fractal dimension, lacunarity, cell area and perimeter characterized an ameboid-like microglial morphology, indicating microglia reactivity in 6-OHDA-WT and 6-OHDA-P2X4KO as compared to shams.

Next, we investigated the ability of P2X4 to modulate microglia reactivity in ADHD-like conditions. For this purpose, we assessed the percentage of changes after 6-OHDA neonatal injection for the four morphological parameters in WT and P2X4KO mice using a two-tailed Unpaired Mann-Whitney test. Differences in cell perimeter, cell area, and fractal dimension were significantly higher in 6-OHDA-WT mice compared to 6-OHDA-P2X4KO mice. No significant difference was detected for lacunarity (Figure 4B2–E2, Supplementary Table S2E). This result showed that the absence of the P2X4 receptor limited neonatal 6-OHDA-induced changes in microglia morphology.

### P2X4 receptor protects against neuroinflammation

Using RT-qPCR, we studied changes in the expression of several cytokines, intracellular markers of neuro-inflammation and glial activation to determine whether the P2X4 receptor affects the response of ACC and PI to neonatal 6-OHDA injection. All values obtained for the ACC and PI are indicated in Supplementary Table S3, S4, respectively. Among all markers tested (see Material and Methods and Supplementary Table S5), only those for which differences between WT and P2X4KO have been detected in ADHD-like conditions are presented. These markers and their possible functions are summarized in Table 1.

**TABLE 1:** List of markers that exhibit expression changes in 6-OHDA-P2X4KO mice in qRT-PCR experiments. They are sorted according to their functions in inflammation, oxidative stress, or intracellular signalling.

	Markers	Type	Function	Cellular expression	References
Secreted Cytokines and Receptors	CCL4	Chemokine	• Chemoattractant for immune regulatory cells (i.e., macrophages, T-lymphocytes)	Astrocytes	Saika et al., 2012a
			• Contributes to neuropathic pain		Sindhu et al., 2019
			• Pro-inflammatory microglial profile		Ullah et al. (2020)
	CCR5	Chemokine receptor	• Chemotaxis of microglia	Microglia Astrocytes	Carbonell et al., 2005
			• Activation of astrocytes and exacerbation of inflammation		Saika et al., 2012b
			• Contributes to neuropathic pain		Liu et al. (2014)
			• Pro-inflammatory microglial profile		
	CX3CL1	Chemokine	• Neuron-microglia communication	Neurons Can be expressed by astrocytes	Lauro et al., 2015, 2019
			• Regulator of microglia activation		Pawelec et al., 2020
			• Can present neuroprotective functions		Silva and Malcangio, 2021
			• Pain-mediating chemokine		Wilkinson et al., 2015
					Yoshida et al. (2001)
	CX3CR1	Chemokine receptor	• Neuron-microglia communication	Microglia	Lee et al., 2018
			• Promote the activation of microglia and stimulate the release of inflammatory factors		Pawelec et al., 2020
			• Pro-inflammatory microglial profile		Tang et al. (2014)
	CXCL12	Chemokine	• Local immune responses in the CNS	Astrocytes Neurons	Guyon, 2014
			• Chemotaxis of leukocytes		Han et al. (2001)
			• Induction of NF-κB signaling pathway		
	IL-16	Cytokine	• Initiating and/or sustaining an inflammatory response	Microglia	Guo et al., 2004
			• Modulates T-cell activation		Little and Cruikshank, 2008
			• Pro-inflammatory microglial profile		Mathy et al. (2000)
	IL-18	Cytokine	• Secretion induced by NLRP3 inflammasome	Microglia Neurons	Alboni et al., 2010
			• Initiation of signaling pathways and inflammatory responses in microglia		Felderhoff-Mueser et al., 2005
			• Exacerbation of neuronal cell death through the increase of Fas-ligand expression		Song et al. (2017)
			• Pro-inflammatory microglial profile		
	IL-6	Cytokine	• Promotes alternative activation of macrophages	Microglia	Codeluppi et al., 2014
			• Inflammatory acute phase response	Astrocytes	Fuster and Walsh, 2014
			• Contributes to both nociceptor and central sensitization	Can be expressed in neurons	Renner et al., 2022
			• Pro-inflammatory and anti-inflammatory microglial profile		Zhou et al. (2016)
	TGF-β	Cytokine	• Suppression of the immune response and glial activation	Microglia Astrocytes	Chen et al., 2015
			• Regulation of astrocyte reactivity		Hisatomi et al., 2002
			• Anti-inflammatory microglial profile		Luo, 2022
					Yoshimura et al. (2010)
	TNF-R	Cytokine receptor	• NF-κB and AP-1 pathway activation	Astrocytes Microglia Neurons	Probert, 2015
			• Role in central pain sensitization		Wajant et al., 2003
			• Pro-inflammatory microglial profile		Zhang et al. (2011)
	TNF-α	Cytokine	• Inflammatory acute phase response	Microglia	Hess et al., 2011
			• NF-κB and AP-1 pathways activation		Idriss and Naismith, 2000
			• Nociceptive responses in the central nervous system		Jang et al., 2021
			• Pro-inflammatory microglial profile		Raffaele et al. (2020)
Intracellular Signaling Markers	GSK3β	Serine/threonine kinase	• Increases cytokine and chemokine production through activation of JNK and NF-κB pathways	Microglia Astrocytes	Abd-Ellah et al., 2018
			• Alterations in GSK3β function are associated with pathological pain		Maixner and Weng, 2013
			• Pro-inflammatory microglial profile		Wang et al. (2010)
	IRF5	Transcription factor	• IRF8-IRF5 transcriptional axis induces P2X4R expression in microglial cells	Microglia	Al Mamun et al., 2020
			• Involved in neuropathic pain		Masuda et al., 2014
			• Pro-inflammatory microglial profile		Tsuda et al. (2017)
	IRF8	Transcription factor	• IRF8-IRF5 transcriptional axis induces P2X4R expression in microglial cells	Microglia	Masuda et al., 2012
			• Contributes to chronic pain		Tsuda et al. (2017)
			• Pro-inflammatory microglial profile		
	NF-κB	Transcription factor	• Induction of NLRP3 inflammasome expression	Microglia Astrocytes Neurons	Babkina et al., 2021
			• Role in inflammatory processes in glial cells (cytokines production, iNOS expression)		Liu et al., 2017
			• Pro-inflammatory microglial profile		Sutterwala et al. (2014)
	NLRP3	Pattern recognition receptor	• Induction of IL-18 and IL-1β secretion through caspase-1 activity	Microglia Can be expressed in neurons and astrocytes	Liu et al., 2017
			• Pro-inflammatory microglial profile		Sutterwala et al., 2014
					Voet et al. (2019)
Astrocytic Markers	GFAP	Class-III intermediate filament	• Marker of reactive astrogliosis	Astrocytes	Amalia, 2021
			• Involved in pro-inflammatory cytokines release		Azzolini et al., 2022
			• Increased expression under inflammatory pain		Ikeda et al. (2013)
Oxidative Stress Markers	Arginase 1	Enzyme	• Downregulation of nitric oxide production by competing with iNOS	Microglia	Cherry et al. (2014)
			• Anti-inflammatory microglial profile		
	SOD1	Enzyme	• Regulates oxidative stress responses	Neurons Astrocytes Microglia	Boillée and Cleveland, 2008
			• Potential anti-inflammatory effects through its reduction of reactive oxygen species		Hwang et al., 2020
			• High SOD1 levels may correlate with postoperative pain reduction		Jaarsma et al., 2008
					KÄRKKÄINEN et al., 2018
					Nagai et al. (2007)
Other Markers	Cathepsin S	Protease	• Role in antigen presentation	Microglia	Hao et al., 2007
			• Role in microglia-neuron communication		Nakagawa et al., 1999
			• Involved in microglia migration towards sites of injury or infection		Pišlar et al., 2021
			• Involved in neuropathic pain		Silva and Malcangio, 2021
					Smyth et al. (2022)
	Iba1	Protein	• Role in actin-crosslinking which is involved in microglial membrane ruffling	Microglia	Hovens et al., 2014
			• Induction of microglial phagocytic activity		Ohsawa et al. (2004)
			• Its increased expression is associated with microglial response		

**FIGURE 5:**
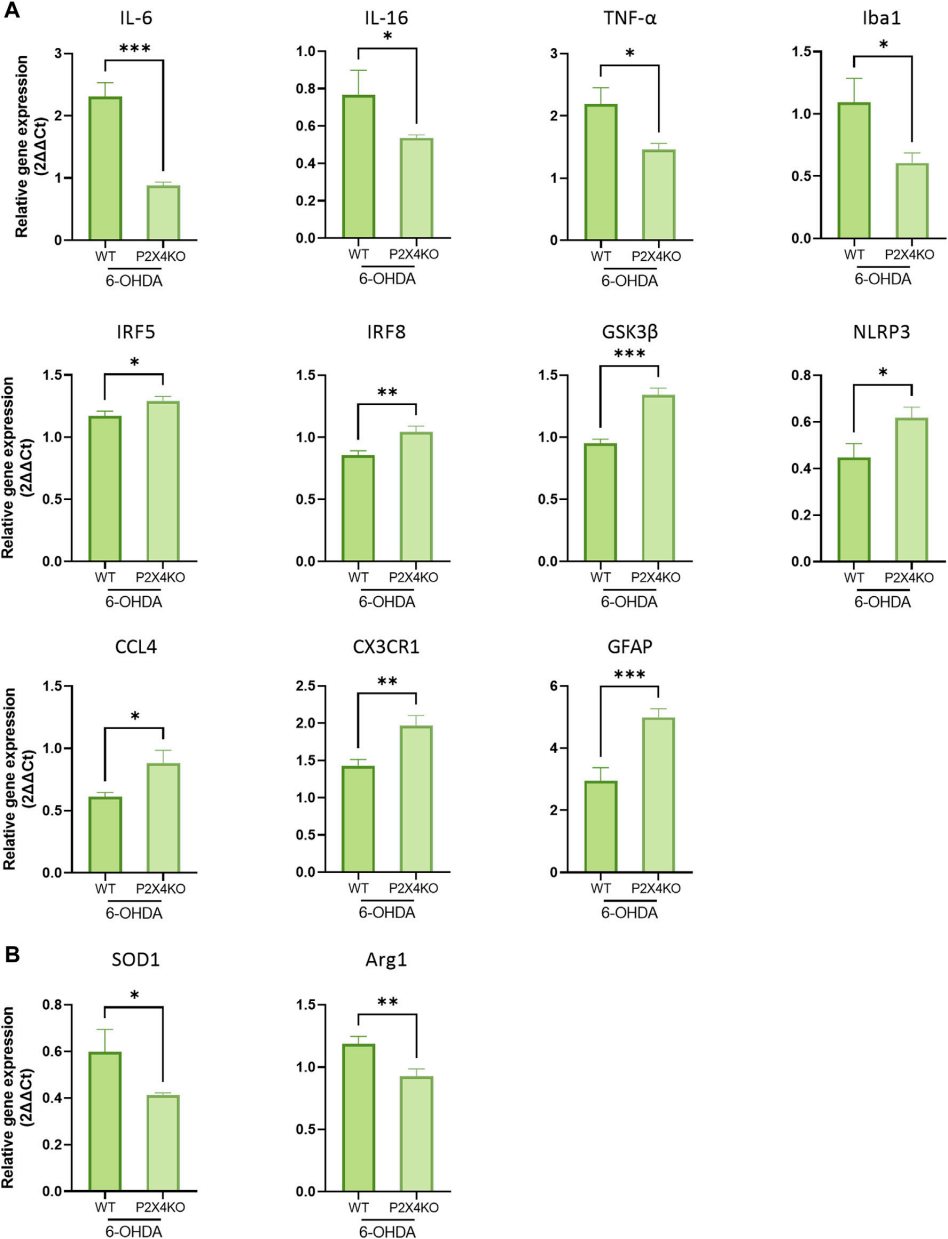
Detection of pro- **(A)** and anti-inflammatory **(B)** markers in the ACC of 6-OHDA-WT and 6-OHDA-P2X4KO mice. All data are expressed as relative gene expression, mean ± SEM (n = 4–11 mice per group), and were analysed with a one-tailed Unpaired *t*-test (*p < 0.05; **p < 0.01; ***p < 0.001).

In the ACC, canonical pro-inflammatory cytokines (IL-6, IL-16, TNF-α) mRNA exhibited decreased amounts in the absence of P2X4. Interestingly, and in agreement with the morphological findings, Iba1 was also expressed at lower levels in 6-OHDA-P2X4KO mice. However, other markers displayed higher levels of expression; most of them being involved in the exacerbation of inflammation (CCL4, CX3CR1, IRF5, and IRF8). Moreover, elevated expression levels of intracellular signalling factors, such as GSK3β and NLRP3, were also detected (Figure 5A, one-tailed Unpaired *t*-test) and may contributed to the amplification of the immune response through the induction of pro-inflammatory cytokines release. The mRNA level of the astrocyte-specific marker GFAP was also significantly increased in P2X4KO mice (Figure 5A). On the contrary, SOD1 and Arg1, two enzymes with protective roles, displayed lower values (Figure 5B, one-tailed Unpaired *t*-test). This finding indicated the presence of an overall predominantly pro-inflammatory microenvironment in the ACC under ADHD-like conditions in both WT and P2X4KO mice.

**FIGURE 6:**
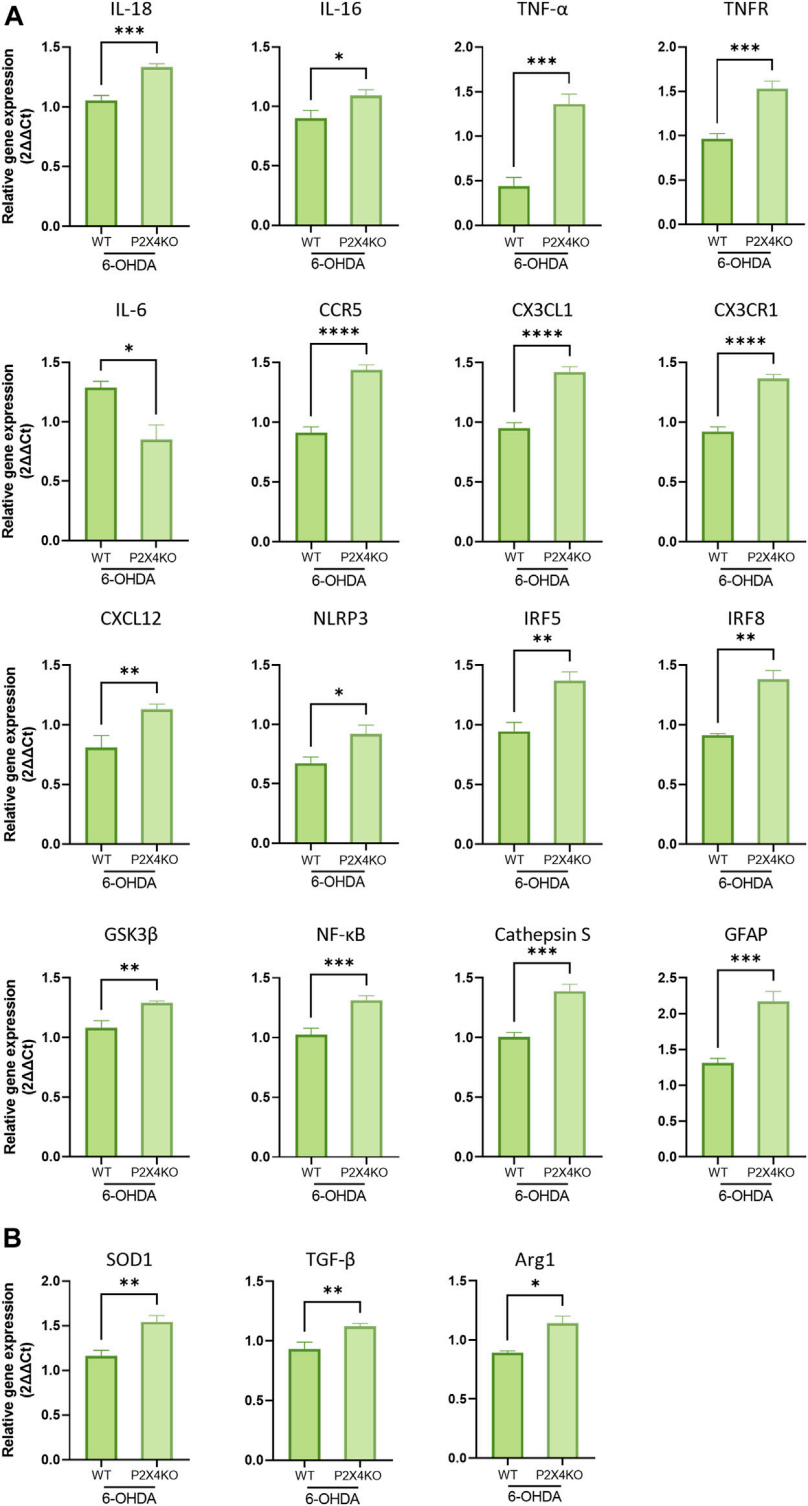
Detection of pro- **(A)** and anti-inflammatory **(B)** markers in the PI of 6-OHDA-WT and 6-OHDA P2X4KO mice. All data are expressed as relative gene expression, mean ± SEM (n = 3–8 mice per group), and were analysed with a two-tailed Unpaired *t*-test (*p < 0.05; **p < 0.01; ***p < 0.001; ****p < 0.0001).

In the PI, the transcripts for secreted or intracellular factors intensifying the immune response were mainly upregulated in 6-OHDA-P2X4KO mice (IL-18, IL-16, TNF-α, TNF-R, CX3CR1). IL-6 mRNA, on the contrary, showed a decreased expression level. Intracellular molecules which mainly lead to a pro-inflammatory microenvironment were also overexpressed (GSK3β, NF-κB, NLRP3, IRF5, and IRF8), along with some chemokines and their receptors (CX3CL1, CXCL12, and CCR5). Astrocytic expression of GFAP was also enhanced, as well as other molecules involved in antigen presentation such as Cathepsin S (Figure 6A, two-tailed Unpaired *t*-test). Interestingly, higher levels of TGF-β, SOD1, and Arg1 mRNA were observed (Figure 6B, two-tailed Unpaired *t*-test). These latter markers are likely to exert an anti-inflammatory role. Taken together, these data pointed towards a predominantly pro-inflammatory microenvironment in the PI under ADHD-like conditions in both WT and P2X4KO mice.

## Discussion

In the present study, we demonstrated that the total deletion of P2X4 prevents neonatal 6-OHDA injection-induced hyperactivity but has only slight effects on pain sensitization that is associated with ADHD-like conditions. Iba1 immunolabeling confirms the activation of microglia in the 6-OHDA mouse model of ADHD. It further indicates that the lack of P2X4 receptor reduces the baseline activation state of microglia and limits, but not suppresses, its reactivity in ADHD-like conditions. Unexpectedly, P2X4 deletion is accompanied by the creation of a proinflammatory environment in the ACC and PI of 6-OHDA adult mice as demonstrated by overexpression of various proinflammatory markers. This situation is further amplified in the ACC by the downregulation of anti-inflammatory molecules.

The 6-OHDA mouse model of ADHD is characterized by alterations of the dopaminergic system that have been extensively considered as a significant factor for the progression of the disease (Albrecht et al., 2015) and may be seen as the primary cause of ADHD pathology (Faraone et al., 2021). Mesocortical dopamine has a pivotal role in selective attention, and working memory, which indicates its involvement in the pathophysiology of ADHD (Levy, 2004; Ruocco et al., 2009). Dopaminergic lesions by neonatal intra-cerebroventricular injection of 6-OHDA in rodents (Sontag et al., 2010) generates ADHD-like models with well-established validity (Avale et al., 2004; Bouchatta et al., 2018). 6-OHDA mice demonstrated good face validity by exhibiting not only the major symptoms of the disease (i.e., hyperactivity, inattention and impulsivity), but also discrete co-existing symptoms (i.e., heightened anxiety, antisocial behaviour and impaired cognitive functions) (Bouchatta et al., 2018; 2020).

Neuroimaging studies in ADHD patients identified structural and functional abnormalities in brain networks with a key role of the ACC (Faraone, 2015) that is at the crossroad of executive functions and pain processing (Garcia-Larrea and Peyron, 2013; Hart et al., 2013; Sellmeijer et al., 2018). We further identified a key role for the ACC-PI pathways in the interaction between ADHD-like conditions and pain sensitization (Bouchatta et al., 2022). Therefore, we focused the present study primarily on the effects of P2X4 deletion in the ACC and on its consequences in the target PI.

ATP release is triggered by inflammatory conditions and in neurological diseases. Its accumulation in the extracellular space regulates synaptic plasticity and neuron-glia interactions through binding to purinergic receptors, including the P2X4 receptor (Lindberg et al., 2015). The effects of P2X4 deletion on microglia phenotype and secretome are instrumental in our understanding of inflammatory processes underlying neurological diseases. Microglia responds to challenges caused by brain diseases by modifying its morphology, molecular profiles and functions. In contrast to the classical, dualist vision of “good” and “bad” microglia, a recent collective update (Paolicelli et al., 2022) made clear that microglia exist in a broad array of highly dynamic, and multidimensional states. This never-resting microglia can be described as a continuum of configurations according to signals from the external and local environments. Disruption of microglia functions has been proposed to account for the pathological onset of neurodevelopmental disorders including ADHD (Bordeleau et al., 2019). Moreover, a genome-wide associated study identified causal genes for ADHD in microglia (Fahira et al., 2019). Accordingly, microglia reactivity in ADHD conditions has been already demonstrated in humans (Yokokura et al., 2021) and animal models (Song et al., 2021; Zhang et al., 2022; Sanches et al., 2023). Recent evidence for microglia reactivity has been also provided in the ACC of the 6-OHDA mouse model (Meseguer-Beltrán et al., 2023).

Over the past decades, the role of P2X4 has been extensively studied in microglia that display abundant expression of this receptor (Tsuda et al., 2003; Vázquez-Villoldo et al., 2014; Duveau et al., 2020). P2X4 has been shown to mediate pain hypersensitivity in the spinal cord of animal models of neuropathic (Tsuda et al., 2003; Coull et al., 2005) and inflammatory (Ulmann et al., 2010; Aby et al., 2018) pain. Reactive microglia may also be a causal agent for ADHD-like symptoms in the spontaneously hypertensive rat model (Zhang et al., 2022), although in other models (i.e., traumatic brain injury-induced ADHD-like conditions) microglia inhibition with minocycline did not affect impulsivity and attention deficits (Pechacek et al., 2022). Although P2X4 was proposed as a male-biased microglial mediator of chronic pain (Halievski et al., 2020), recent findings showed that microglial P2X4 is crucial for neuropathic pain, regardless of sex (Gilabert et al., 2023). In agreement, sex comparisons in WT and P2X4KO mice did not reveal any differences between males and females in hyperactivity, impulsivity or sensory thresholds.

Interestingly, the present study demonstrates an unexpected function of P2X4 that may contribute to basal microglia homeostasis. Indeed, its deletion amplifies the features of homeostatic microglia in sham conditions. In 6-OHDA mice, P2X4 suppression attenuates but does not fully prevent microglia reactivity. In addition, our findings indicate a less reactive microglia phenotype in 6-OHDA-P2X4KO than in 6-OHDA-WT. Beside the morphological analysis, the decreased reactive phenotype of ACC microglia in ADHD-like conditions is also characterized in our study by the decreased expression of i) the microglia-specific marker Iba1, and ii) the IL16 and TNF-α cytokines that are mostly released by microglial cells (Borst et al., 2021). Limited microglia reactivity in 6-OHDA-P2X4KO mice may explain the lack of hyperactivity (distance travelled) and impulsivity-like (velocity) behaviours induced by ADHD-like conditions in P2X4KO mice.

Despite this reduced microglia reactivity, the qRT-PCR analysis of cytokine expression in ADHD-like conditions suggest that P2X4 deletion favours a predominantly pro-inflammatory environment in the ACC and PI as compared to WT mice. Table 1 recapitulates these markers sorted according to their possible functions. Microglia pro-inflammatory cytokine receptor (CX3CR1), and transcription factors (IRF5 and IRF8) are upregulated in the ACC and PI of 6-OHDA-P2X4KO mice. The expression of pro-inflammatory markers of intracellular signalling pathways is also increased in both regions (GSK3β, NLRP3, NF-κB). The overexpression of astrocytic markers (GFAP) (Giovannoni and Quintana, 2020) and chemokines (CCL4 in the ACC, CXCL12 in the PI) (Guyon, 2014; Zhu et al., 2014) are indicative of astrogliosis that may also contribute to the pro-inflammatory environment.

The results displayed striking differences in the two brain regions investigated. The pro-inflammatory environment is more pronounced in the PI than in the ACC. As an example, IL18 is overexpressed in the PI while no changes were noticed in the ACC. Interestingly, its release is stimulated by NLRP3 (Amores-Iniesta et al., 2017) that is also upregulated in the PI of 6-OHDA-P2X4KO mice. Modifications of cytokine receptors expression were also detected in the PI (TNFR, CCR5).

Other markers did not show any significant difference in their levels of expression following the deletion of the P2X4 receptor under ADHD-like conditions. Remarkably, some of these molecules were previously shown to be involved in neuropathic pain (MMP9, BDNF) (Kawasaki et al., 2008; Thakkar and Acevedo, 2023) or pain pathogenesis (Wnt5a) (Wang et al., 2020; Liu et al., 2023). These results are in line with the absence of effects of P2X4 deletion on pain sensitization.

Many inflammatory pathways exist in neural cells and some changes in the inflammatory environment may be indirectly triggered by P2X4 deletion. P2X7 is another purinergic receptor known to play prominent roles in inflammatory processes (Di Virgilio et al., 2017) by promoting cytokine production by microglia and astrocytes (Sperlágh and Illes, 2014). P2X7 interacts with P2X4 (Schneider et al., 2017) and P2X4 deletion may thus impact purinergic signalling at large, beyond the sole P2X4 receptor.

The mild pro-inflammatory environment of 6-OHDA-P2X4KO mice is associated with the prevention of hyperactivity/impulsivity and might exert a protective role against ADHD-like symptoms. However, the various pro-inflammatory features described in the 6-OHDA-P2X4KO vs. sham mice are likely to have different, possibly opposite, consequences. Inflammation-induced astrocyte reactivity has been implicated in ADHD-like conditions (Sandau et al., 2012; Zhang et al., 2022; Liu et al., 2023) while in our model it is associated with reduced symptoms. In contrast, a protective role is in agreement with the amplified homeostatic features of microglia observed in P2X4KO mice. In several cases, P2X4 contributes to the development of neurological disorders through well-identified mechanisms, e.g., in hippocampal neurons for Alzheimer’s disease (Varma et al., 2009), or in spinal microglia for neuropathic pain (Coull et al., 2005). Conversely, protective roles of P2X4 have been described on motoneurons in amyotrophic lateral sclerosis (Andries et al., 2007) and autoimmune encephalitis (Zabala et al., 2018). In line with our results, the latter study demonstrated an increase in pro-inflammatory gene expression in a P2X4KO mouse model of autoimmune encephalitis as compared to WT mice. In contrast to its effects on hyperactivity and impulsivity, P2X4 deletion does not change mechanical or thermal hypersensitivity in 6-OHDA mice. P2X4 deletion may even induce basal thermal hypersensitivity in sham-P2X4KO mice as compared to WT animals.

The dual effect of P2X4 deletion on ADHD-like symptoms and pain sensitization could result from the dysfunction of distinct cell types. P2X4 is expressed in both neurons and microglia (Bo et al., 2003) and plays different functions in these 2two cell populations (Duveau et al., 2020). Beneficial impact of both overexpression or deletion of P2X4 described in SOD1 mouse model of amyotrophic lateral sclerosis suggests that P2X4 expressed in motoneurons, and microglia exert opposite effects in ALS pathogenesis (Bertin et al., 2022). In addition, compelling evidence indicates the existence of distinct microglia subpopulations (Stratoulias et al., 2023) that may differ with regard to the expression and/or function of P2X4. The comorbidity of ADHD-like symptoms and pain sensitization may be underpinned by different cell types and/or by the dysfunction of different brain areas.

Our data revealed a complex role of P2X4 likely dependent on a combination of glial and neuronal effects. The lack of P2X4 triggers the development of predominantly pro-inflammatory but distinct environments in the ACC and PI of 6-OHDA mice. In ADHD-like conditions, it decreases microglial reactivity in the ACC and alleviates hyperactivity/impulsivity while only slight alterations of nociceptive behavior are observed. P2X4 deletion has apparent contradictory effects, amplifying the homeostatic characteristics of ACC microglia but promoting the over-expression of pro-inflammatory markers. Importantly, however, these results confirm that microglia activation and inflammation are not equivalent (Paolicelli et al., 2022). In ADHD-like conditions, the lack of P2X4 modulates the microglia phenotype and modifies the “cytokine-enriched secretomes” from microglia and most probably from other cell types as well.

Taken together, our findings suggest that P2X4 facilitates microglia reactivity but at the same time exerts a protective role on the expression of some pro-inflammatory markers, possibly from non-microglia origin. This dual role of P2X4 could be responsible for the differential effects noted on ADHD-like symptoms and pain sensitization.

## Data Availability

The datasets presented in this study can be found in online repositories. The names of the repository/repositories and accession number(s) can be found below: https://github.com/marclandry33/p2x4-adhd.
