# Supplementary material for: P2X4 signalling contributes to hyperactivity but not pain sensitization comorbidity in a mouse model of attention deficit/hyperactivity disorder
Source: Front Pharmacol. 2024 Jan 4;14:1288994. doi: 10.3389/fphar.2023.1288994 (PMC10794506; doi:10.3389/fphar.2023.1288994)
Supplement: Supplementary file 1 [file Table1.DOCX]

**Table S1**

| **FEMALES** | | | | | | | | | | | |
| --- | --- | --- | --- | --- | --- | --- | --- | --- | --- | --- | --- |
| **Behavioural parameter** | **Sham-WT Mice** | | **Sham-P2X4KO Mice** | | **6-OHDA-WT Mice** | | **6-OHDA-P2X4KO Mice** | | **Comparison** | ***t* value** | **p**  **value** |
|  | **Mean ± SEM** | **Number of mice** | **Mean ± SEM** | **Number of mice** | **Mean ± SEM** | **Number of mice** | **Mean ± SEM** | **Number of mice** |  |  |  |
| **Distance travelled (cm)** | 3484 ± 165.1 | 10 | 3538 ± 211.8 | 10 | 4612 ± 337 | 18 | 3907 ± 396 | 7 | Sham-WT  vs  6-OHDA -WT | t=2.391, df=26 | p=0.0243 |
|  |  |  |  |  |  |  |  |  | Sham-P2X4KO  vs  6-OHDA -P2X4KO | t=0.889, df=15 | p=0.3883 |
|  |  |  |  |  |  |  |  |  | Sham-WT  vs  Sham-P2X4KO | t=0.2034, df=18 | p=0.8411 |
|  |  |  |  |  |  |  |  |  | 6-OHDA-WT  vs  6-OHDA-P2X4KO | t=1.180, df=23 | p=0.2500 |
| **Velocity (cm/s)** | 6.28 ± 0.41 | 10 | 5.75 ± 0.44 | 10 | 8.35 ± 0.57 | 18 | 6.26 ± 0.73 | 7 | Sham-WT  vs  6-OHDA -WT | t=2.512, df=26 | p=0.0186 |
|  |  |  |  |  |  |  |  |  | Sham-P2X4KO  vs  6-OHDA -P2X4KO | t=0.631, df=15 | p=0.5374 |
|  |  |  |  |  |  |  |  |  | Sham-WT  vs  Sham-P2X4KO | t=0.8651, df=18 | p=0.3983 |
|  |  |  |  |  |  |  |  |  | 6-OHDA-WT  vs  6-OHDA-P2X4KO | t=2.045, df=23 | p=0.0525 |

**A**

**B**

| **MALES** | | | | | | | | | | | |
| --- | --- | --- | --- | --- | --- | --- | --- | --- | --- | --- | --- |
| **Behavioural parameter** | **Sham-WT Mice** | | **Sham-P2X4KO Mice** | | **6-OHDA-WT Mice** | | **6-OHDA-P2X4KO Mice** | | **Comparison** | ***t* value** | **p**  **value** |
|  | **Mean ± SEM** | **Number of mice** | **Mean ± SEM** | **Number of mice** | **Mean ± SEM** | **Number of mice** | **Mean ± SEM** | **Number of mice** |  |  |  |
| **Distance travelled (cm)** | 3727 ± 197.7 | 11 | 3873 ± 291.9 | 8 | 4793 ± 283.3 | 9 | 4304 ± 240.6 | 12 | Sham-WT  vs  6-OHDA -WT | t=3.169, df=18 | p=0.0053 |
|  |  |  |  |  |  |  |  |  | Sham-P2X4KO  vs  6-OHDA -P2X4KO | t=1.137, df=18 | p=0.2703 |
|  |  |  |  |  |  |  |  |  | Sham-WT  vs  Sham-P2X4KO | t=0.4288, df=17 | p=0.6734 |
|  |  |  |  |  |  |  |  |  | 6-OHDA-WT  vs  6-OHDA-P2X4KO | t=1.320, df=19 | p=0.2025 |
| **Velocity (cm/s)** | 6.67 ± 0.26 | 11 | 6.94  ± 0.55 | 8 | 8.36 ± 0.69 | 9 | 6.97 ± 0.42 | 12 | Sham-WT  vs  6-OHDA -WT | t=2.468, df=18 | p=0.0238 |
|  |  |  |  |  |  |  |  |  | Sham-P2X4KO  vs  6-OHDA -P2X4KO | t=0.044, df=18 | p=0.9651 |
|  |  |  |  |  |  |  |  |  | Sham-WT  vs  Sham-P2X4KO | t=0.4749, df=17 | p=0.6409 |
|  |  |  |  |  |  |  |  |  | 6-OHDA-WT  vs  6-OHDA-P2X4KO | t=1.825, df=19 | p=0.0838 |

| **FEMALES** | | | | | | | | | | | |
| --- | --- | --- | --- | --- | --- | --- | --- | --- | --- | --- | --- |
| **Behavioural parameter** | **Sham-WT Mice** | | **Sham-P2X4KO Mice** | | **6-OHDA-WT Mice** | | **6-OHDA-P2X4KO Mice** | | **Comparison** | ***U* value** | **p**  **value** |
|  | **Mean ± SEM** | **Number of mice** | **Mean ± SEM** | **Number of mice** | **Mean ± SEM** | **Number of mice** | **Mean ± SEM** | **Number of mice** |  |  |  |
| **Mechanical pain threshold (g)** | 1.14 ± 0.05 | 11 | 1.22 ± 0.03 | 10 | 0.66 ± 0.05 | 17 | 0.57 ± 0.09 | 7 | Sham-WT  vs  6-OHDA -WT | U=9 | p=0.0006x10^-2^ |
|  |  |  |  |  |  |  |  |  | Sham-P2X4KO  vs  6-OHDA -P2X4KO | U=0.50 | p=0.0001 |
|  |  |  |  |  |  |  |  |  | Sham-WT  vs  Sham-P2X4KO | U=39 | p=0.2119 |
|  |  |  |  |  |  |  |  |  | 6-OHDA-WT  vs  6-OHDA-P2X4KO | U=41.50 | p=0.2528 |
| **Amplitude of changes in mechanical threshold**  **(% of sham)** |  |  |  |  | -41.74 ± 4.38 | 17 | -53.67 % ± 7.45 | 7 | 6-OHDA-WT  vs  6-OHDA-P2X4KO | U=30 | p=0.0592 |

**C**

| **MALES** | | | | | | | | | | | |
| --- | --- | --- | --- | --- | --- | --- | --- | --- | --- | --- | --- |
| **Behavioural parameter** | **Sham-WT Mice** | | **Sham-P2X4KO Mice** | | **6-OHDA-WT Mice** | | **6-OHDA-P2X4KO Mice** | | **Comparison** | ***U* value** | **p**  **value** |
|  | **Mean ± SEM** | **Number of mice** | **Mean ± SEM** | **Number of mice** | **Mean ± SEM** | **Number of mice** | **Mean ± SEM** | **Number of mice** |  |  |  |
| **Mechanical pain threshold (g)** | 1.13 ± 0.05 | 11 | 1.25 ± 0.05 | 8 | 0.75 ± 0.04 | 9 | 0.71 ± 0.09 | 12 | Sham-WT  vs  6-OHDA -WT | U=3 | p=0.0042x10^-2^ |
|  |  |  |  |  |  |  |  |  | Sham-P2X4KO  vs  6-OHDA -P2X4KO | U=7 | p=0.0651x10^-2^ |
|  |  |  |  |  |  |  |  |  | Sham-WT  vs  Sham-P2X4KO | U=26.50 | p=0.1437 |
|  |  |  |  |  |  |  |  |  | 6-OHDA-WT  vs  6-OHDA-P2X4KO | U=43.50 | p=0.4680 |
| **Amplitude of changes in mechanical threshold**  **(% of sham)** |  |  |  |  | -33.78 ± 3.90 | 9 | -42.57 ± 7.50 | 12 | 6-OHDA-WT  vs  6-OHDA-P2X4KO | U=40 | p=0.3285 |

**D**

| **FEMALES** | | | | | | | | | | | |
| --- | --- | --- | --- | --- | --- | --- | --- | --- | --- | --- | --- |
| **Behavioural parameter** | **Sham-WT Mice** | | **Sham-P2X4KO Mice** | | **6-OHDA-WT Mice** | | **6-OHDA-P2X4KO Mice** | | **Comparison** | ***t* value** | **p**  **value** |
|  | **Mean ± SEM** | **Number of mice** | **Mean ± SEM** | **Number of mice** | **Mean ± SEM** | **Number of mice** | **Mean ± SEM** | **Number of mice** |  |  |  |
| **Thermal pain threshold (sec)** | 8.07 ± 0.26 | 11 | 6.78 ± 0.30 | 10 | 5.29 ± 0.26 | 16 | 5.62 ± 0.18 | 7 | Sham-WT  vs  6-OHDA -WT | t=7.145, df=25 | p=0.1729x10^-6^ |
|  |  |  |  |  |  |  |  |  | Sham-P2X4KO  vs  6-OHDA -P2X4KO | t=2.994, df=15 | p=0.0091 |
|  |  |  |  |  |  |  |  |  | Sham-WT  vs  Sham-P2X4KO | t=3.238, df=19 | p=0.0043 |
|  |  |  |  |  |  |  |  |  | 6-OHDA-WT  vs  6-OHDA-P2X4KO | t=0.767, df=21 | p=0.4517 |
| **Amplitude of changes in thermal threshold (% of sham)** |  |  |  |  | -36.90 ± 3.10 | 16 | -21.80 ± 2.50 | 7 | 6-OHDA-WT  vs  6-OHDA-P2X4KO | t=3.009, df=21 | p=0.0067 |

**E**

| **MALES** | | | | | | | | | | | |
| --- | --- | --- | --- | --- | --- | --- | --- | --- | --- | --- | --- |
| **Behavioural parameter** | **Sham-WT Mice** | | **Sham-P2X4KO Mice** | | **6-OHDA-WT Mice** | | **6-OHDA-P2X4KO Mice** | | **Comparison** | ***t* value** | **p**  **value** |
|  | **Mean ± SEM** | **Number of mice** | **Mean ± SEM** | **Number of mice** | **Mean ± SEM** | **Number of mice** | **Mean ± SEM** | **Number of mice** |  |  |  |
| **Thermal pain threshold (sec)** | 8.93 ± 0.39 | 10 | 7.69 ± 0.33 | 8 | 5.94 ± 0.39 | 9 | 5.96 ± 0.23 | 12 | Sham-WT  vs  6-OHDA -WT | t=5.373, df=17 | p=0.0050x10^-2^ |
|  |  |  |  |  |  |  |  |  | Sham-P2X4KO  vs  6-OHDA -P2X4KO | t=4.422, df=18 | p=0.0003 |
|  |  |  |  |  |  |  |  |  | Sham-WT  vs  Sham-P2X4KO | t=2.346, df=16 | p=0.0322 |
|  |  |  |  |  |  |  |  |  | 6-OHDA-WT  vs  6-OHDA-P2X4KO | t=0.046, df=19 | p=0.9634 |
| **Amplitude of changes in thermal threshold (% of sham)** |  |  |  |  | -29.29 ± 4.68 | 9 | -16.84 ± 3.26 | 12 | 6-OHDA-WT  vs  6-OHDA-P2X4KO | t=2.252, df=19 | p=0.0364 |

**F**

| **FEMALES vs MALES** | | | | | | | | | | | | |
| --- | --- | --- | --- | --- | --- | --- | --- | --- | --- | --- | --- | --- |
| **Experimental group** | **Distance travelled (cm)** | | **Velocity (cm/s)** | | **Mechanical pain threshold (g)** | | **Amplitude of changes in mechanical threshold**  **(% of sham)** | | **Thermal pain threshold (sec)** | | **Amplitude of changes in thermal threshold (% of sham)** | |
| **Sham-WT** | ***t* value** | t=0.9352, df=19 | ***t* value** | t=0.8198, df=19 | ***U* value** | U=59.50 |  | | ***t* value** | t=1.849, df=19 |  | |
|  | **p value** | p=0.3614 | **p value** | p=0.4225 | **p value** | p=0.9910 |  |  | **p value** | p=0.0800 |  |  |
| **6-OHDA-WT** | ***t* value** | t=0.3495, df=25 | ***t* value** | t=0.01570, df=25 | ***U* value** | U=52.50 | ***U* value** | U=52.50 | ***t* value** | t=1.408, df=23 | ***t* value** | t=1.405, df=23 |
|  | **p value** | p=0.7296 | **p value** | p=0.9876 | **p value** | p=0.1899 | **p value** | p=0.1899 | **p value** | p=0.1726 | **p value** | p=0.1734 |
| **Sham-P2X4KO** | ***t* value** | t=0.9505, df=16 | ***t* value** | t=1.698, df=16 | ***U* value** | U=34.50 |  | | ***t* value** | t=2.043, df=16 |  | |
|  | **p value** | p=0.3560 | **p value** | p=0.1089 | **p value** | p=0.6078 |  |  | **p value** | p=0.0579 |  |  |
| **6-OHDA-P2X4KO** | ***t* value** | t=0.9127, df=17 | ***t* value** | t=0.9097, df=17 | ***U* value** | U=30.50 | ***t* value** | U=30.50 | ***t* value** | t=1.026, df=17 | ***t* value** | t=1.051, df=17 |
|  | **p value** | p=0.3741 | **p value** | p=0.3757 | **p value** | p=0.3258 | **p value** | p=0.3258 | **p value** | p=0.3195 | **p value** | p=0.3079 |

**G**
