# Supplementary material for: P2X4 signalling contributes to hyperactivity but not pain sensitization comorbidity in a mouse model of attention deficit/hyperactivity disorder
Source: Front Pharmacol. 2024 Jan 4;14:1288994. doi: 10.3389/fphar.2023.1288994 (PMC10794506; doi:10.3389/fphar.2023.1288994)
Supplement: Supplementary file 2 [file Table2.DOCX]

**Table S2**

| **Morphological parameter** | **Sham-WT Mice** | | **Sham-P2X4KO Mice** | | ***U* value** | **p-value** |
| --- | --- | --- | --- | --- | --- | --- |
|  | **Mean ± SEM** | **Number of mice** | **Mean ± SEM** | **Number of mice** |  |  |
| **Cell perimeter (μm)** | 145.5 ± 1.29 | 7 | 214.4 ± 2.20 | 6 | U=101072 | p<10^-15^ |
| **Cell area (μm^2^)** | 1389 ± 25.63 | 7 | 3234 ± 68.13 | 6 | U=91723 | p<10^-15^ |
| **Fractal dimension** | 1.43 ± 0.01 | 7 | 1.49 ± 0.01 | 6 | U=138811 | p<10^-15^ |
| **Lacunarity** | 0.23 ± 0.01 | 7 | 0.27 ± 0.01 | 6 | U=151845 | p<10^-15^ |

**A**

**B**

| **Morphological parameter** | **6-OHDA-WT Mice** | | **6-OHDA-P2X4KO Mice** | | ***U* value** | **p-value** |
| --- | --- | --- | --- | --- | --- | --- |
|  | **Mean ± SEM** | **Number of mice** | **Mean ± SEM** | **Number of mice** |  |  |
| **Cell perimeter (μm)** | 124 ± 0.98 | 6 | 212.8 ± 2.24 | 7 | U=26467 | p<10^-15^ |
| **Cell area (μm^2^)** | 993.8 ± 16.77 | 6 | 3067 ± 61.07 | 7 | U=25851 | p<10^-15^ |
| **Fractal dimension** | 1.40 ± 0.01 | 6 | 1.49 ± 0.01 | 7 | U=96876 | p<10^-15^ |
| **Lacunarity** | 0.22 ± 0.01 | 6 | 0.27 ± 0.01 | 7 | U=91268 | p<10^-15^ |

**C**

| **Morphological parameter** | **Sham-WT Mice** | | **6-OHDA-WT Mice** | | ***U* value** | **p-value** |
| --- | --- | --- | --- | --- | --- | --- |
|  | **Mean ± SEM** | **Number of mice** | **Mean ± SEM** | **Number of mice** |  |  |
| **Cell perimeter (μm)** | 145.5 ± 1.29 | 7 | 124 ± 0.98 | 6 | U=308192 | p<10^-15^ |
| **Cell area (μm^2^)** | 1389 ± 25.63 | 7 | 993.8 ± 16.77 | 6 | U=307115 | p<10^-15^ |
| **Fractal dimension** | 1.46 ± 0.01 | 7 | 1.340 ± 0.01 | 6 | U=374779 | p= 1.3x10^-11^ |
| **Lacunarity** | 0.23 ± 0.01 | 7 | 0.22 ± 0.01 | 6 | U=368254 | p= 2.8×10^-13^ |

| **Morphological parameter** | **Sham-P2X4KO Mice** | | **6-OHDA-P2X4KO Mice** | | ***U* value** | **p-value** |
| --- | --- | --- | --- | --- | --- | --- |
|  | **Mean ± SEM** | **Number of mice** | **Mean ± SEM** | **Number of mice** |  |  |
| **Cell perimeter (μm)** | 214.4 ± 2.20 | 6 | 212.8 ± 2.24 | 7 | U=138446 | p=0.0445 |
| **Cell area (μm^2^)** | 3234 ± 68.13 | 6 | 3067 ± 61.07 | 7 | U=138329 | p=0.0422 |
| **Fractal dimension** | 1.49 ± 0.01 | 6 | 1.48 ± 0.01 | 7 | U=145976 | p=0.5557 |
| **Lacunarity** | 0.27 ± 0.01 | 6 | 0.26 ± 0.003 | 7 | U=135478 | p=0.0122 |

**D**

**E**

| **Morphological parameter (percentage of changes)** | **6-OHDA-WT Mice** | | **6-OHDA-P2X4KO Mice** | | ***U* value** | **p-value** |
| --- | --- | --- | --- | --- | --- | --- |
|  | **Mean ± SEM** | **Number of mice** | **Mean ± SEM** | **Number of mice** |  |  |
| **Cell perimeter (%)** | -14.77 ± 0.67 | 6 | -0.73 ± 1.05 | 7 | U=129461 | p<10^-15^ |
| **Cell area (%)** | -28.45 ± 1.21 | 6 | -5.18 ± 1.89 | 7 | U= 129257 | p<10^-15^ |
| **Fractal dimension (%)** | -1.83 ± 0.22 | 6 | -0.57 ± 0.27 | 7 | U=189150 | p=0.0005 |
| **Lacunarity (%)** | -5.38 ± 0.39 | 6 | -3.72 ± 0.81 | 7 | U= 190099 | p=0.5299 |
