# Supplementary material for: P2X4 signalling contributes to hyperactivity but not pain sensitization comorbidity in a mouse model of attention deficit/hyperactivity disorder
Source: Front Pharmacol. 2024 Jan 4;14:1288994. doi: 10.3389/fphar.2023.1288994 (PMC10794506; doi:10.3389/fphar.2023.1288994)
Supplement: Supplementary file 3 [file Table5.DOCX]

**Table S5**

| **Gene** | **Forward Primer** | **Reverse Primer** |
| --- | --- | --- |
| APE1 | GGTTGGCGCCTTGATTACTTT | GACCGGATCTTGCTGTCACAC |
| **Arg1** | **AACACGGCAGTGGCTTTAACC** | **GGTTTTCATGTGGCGCATTC** |
| BDNF | CCCGTCTGTACTTTACCCTTTGG | TGACTAGGGAAATGGGCTTAACA |
| **Cathepsin S** | **GGCAAAGATTACTGGCTTGTGA** | **GCCATCCGAATGTATCCTTGAT** |
| CCL2 | AGGTGTCCCAAAGAAGCTGT | ATGTCTGGACCCATTCCTTC |
| CCL4 | CTCCCGGCAGCTTCACAGA | GCTGGCTTGGAGCAAAGACT |
| CCR2 | TGCAACTGCCTCTTTCCTCAT | TTGGCTATTCCATATACACCTTTCC |
| **CCR5** | **TACTCGGCTTCTAGTCAGGCATT** | **GTGAGAGGAGGGAGCTTTGTGT** |
| CD11b | CTCATCACTGCTGGCCTATACAA | GCAGCTTCATTCATCATGTCCTT |
| **CX3CL1** | **CATGCTCCACAGAACCAGTTGT** | **CACCGAAGGAGCCAGGATATAGT** |
| **CX3CR1** | **GTCTGTATGTTTGTGTCGAGGATGA** | **ACAAAGGGTTGGATATGCCTGT** |
| CXCL1 | GGTAGGGCATAATGCCCTTTTA | CGTCTCTGTCCCGAGCGA |
| **CXCL12** | **CAGGCCGTGCACCCTTT** | **ATGGGCCAGGGTAGCTGTT** |
| CXCL13 | TCGAGGAATGAAAAACCTACATGTC | TCCATCTCGCAAACCTCTTGTT |
| CXCR2 | GCTGTCTCCTGAGGAGCTCT | CTCTATCCAATGGATGATTGTGA |
| CXCR5 | GCAAGATAGCAAAGTGGTCCTAGC | AGGAACCTCTGTCGTCATTCTCTTA |
| **GFAP** | **TTTCTCAACCTCCAGATCC** | **CCGCATCTCCACAGTCTTTA** |
| **GSK3β** | **GCCTTCAGCTTTTGGTAGCAT** | **TGCCACTACTGTGGTTACCTT** |
| **Iba1** | **CAATTCCTCGATGATCCCAAATA** | **TTCACCTTGAAGGCTTCAAGTTT** |
| IL-10 | AGTTGTGAAGAAACTCATGGGTCTT | TGCTGCAGGAATGATCATCAA |
| IL-13 | GAAGAATGGCCTGTTACACTCAAG | TGTTCAGTGACAAACCCACCAC |
| **IL-16** | **GCCATGAAGAACACATTCAAACA** | **TGGTTCCACTCCTTCTAGGCTCT** |
| **IL-18** | **ATTAGCACACATGCGCCTTGT** | **AAATCATGCAGCCTCGGGTA** |
| IL-1β | GAAGAAGAGCCCATCCTCTG | TCATCTCGGAGCCTGTAGTG |
| IL-2 | ACTAAAGGGCTCTGACAACACATTT | TCAGAAAGTCCACCACAGTTGCT |
| IL-4 | ATCATCGGCATTTTGAACGAG | AGGACGTTTGGCACATCCAT |
| **IL-6** | **TACTCGGCAAACCTAGTGCGT** | **ATTTTCTGACCACAGTGAGGAATG** |
| INF-γ | ACAATGAACGCTACACACTGCAT | TGGCAGTAACAGCCAGAAACAG |
| **IRF5** | **CCTGGGACAGCCACGTAGAC** | **CAGAACCCTGTAGCCAGCCA** |
| **IRF8** | **CAGCTGTCAATCACTTGAGA** | **CAAGCAGTATCTACACCCATATT** |
| MMP9 | TTGCCCCTACTGGAAGGTATTATGT | GAGTGGATAGCTCGGTGGTGTT |
| **NF-κB** | **CAAAGCCCTGACAGTCCATTG** | **GATTCTCCAGCACCTTTGGATT** |
| **NLRP3** | **ATAGGGTCTGGAGCAAAGGCTT** | **TCTGGCAGTTTCACGTTATCGA** |
| NOX2 | TTTCCTGTGTGGCCCTGAA | GGCCGGATTCTGAGTTGGA |
| P2X4R | TTGGCTGGGTCACCTGTTG | CGTGTCTCTGCTCCCATATTCC |
| Pecam1 | CGAGAGTCCTGTGCACGTATTT | TGGATGGCTTGGCCTGAA |
| Plat (tPA) | AAGATCATGTCTCAACAGCAAAAAA | TCTTGTCCCCAGTGCAAACTTT |
| **SOD1** | **CCCGGCGGATGAAGAGA** | **CATTGGCCACACCGTCCT** |
| **TGF-β** | **CATGCCAACTTCTGTCTGGGA** | **GCAAGGACCTTGCTGTACTGTGT** |
| TLR7 | GGAAGAGACTCTGCAGGAGCTC | CACTGCCAGAAGTATGGGTGAG |
| **TNF-R** | **AAGAAATGTCCCAGGTGGAG** | **TCTCACTCAGGTAGCGTTGG** |
| **TNF-α** | **GGCACTCCCCCAAAAGATG** | **GCCACAAGCAGGAATGAGAAG** |
| TSPO | TGGCTCCTACATAGTCTGGAAAGA | TGTAGAGACCCAAGGGAACCAT |
| Wnt3a | tcggatacctcttagtgctct | ggggtcccacagccaagga |
| Wnt5a | ccagttccggcatcggagat | ggcgttcaccaccccagct |
