# Supplementary material for: P2X4 signalling contributes to hyperactivity but not pain sensitization comorbidity in a mouse model of attention deficit/hyperactivity disorder
Source: Front Pharmacol. 2024 Jan 4;14:1288994. doi: 10.3389/fphar.2023.1288994 (PMC10794506; doi:10.3389/fphar.2023.1288994)
Supplement: Supplementary file 4 [file Table3.DOCX]

| **Marker** | **6-OHDA-WT Mice** | | **6-OHDA-P2X4KO Mice** | | ***t* test value** | **p-value** |
| --- | --- | --- | --- | --- | --- | --- |
|  | **Mean ± SEM** | **Number of mice** | **Mean ± SEM** | **Number of mice** |  |  |
| **IL-6** | 2.31 ± 0.23 | 4 | 0.89 ± 0.06 | 5 | t=6.827; df=7 | p=0.0001 |
| **IL-16** | 0.77 ± 0.13 | 5 | 0.54 ± 0.02 | 7 | t=2.112; df=10 | P=0.0304 |
| **TNF-α** | 2.20 ± 0.26 | 4 | 1.46 ± 0.10 | 5 | t=2.920; df=7 | p=0.0112 |
| **Iba1** | 1.09 ± 0.19 | 11 | 0.61 ± 0.08 | 9 | t=2.164; df=18 | p=0.0221 |
| **CX3CR1** | 1.43 ± 0.09 | 7 | 1.97 ± 0.13 | 8 | t=3.319; df=13 | p=0.0028 |
| **IRF5** | 1.17 ± 0.04 | 5 | 1.29 ± 0.04 | 8 | t=2.174; df=11 | p=0.0262 |
| **IRF8** | 0.85 ± 0.04 | 5 | 1.05 ± 0.05 | 8 | t=2.903; df=11 | p=0.0072 |
| **CCL4** | 0.61 ± 0.04 | 4 | 0.88 ± 0.10 | 8 | t=1.828; df=10 | P=0.0487 |
| **GSK3β** | 0.95 ± 0.04 | 5 | 1.34 ± 0.05 | 8 | t=5.274; df=11 | p=0.0001 |
| **NLRP3** | 0.45 ± 0.06 | 4 | 0.62 ± 0.05 | 8 | t=2.180; df=10 | p=0.0271 |
| **GFAP** | 2.947 ± 0.427 | 7 | 4.99 ± 0.28 | 8 | t=4.113; df=13 | p=0.0006 |
| **SOD1** | 0.60 ± 0.10 | 8 | 0.41 ± 0.01 | 8 | t=1.895; df=14 | p=0.0395 |
| **Arg1** | 1.19 ± 0.06 | 6 | 0.93 ± 0.06 | 8 | t=2.961; df=12 | p=0.0059 |

**Table S3**
