# Supplementary material for: P2X4 signalling contributes to hyperactivity but not pain sensitization comorbidity in a mouse model of attention deficit/hyperactivity disorder
Source: Front Pharmacol. 2024 Jan 4;14:1288994. doi: 10.3389/fphar.2023.1288994 (PMC10794506; doi:10.3389/fphar.2023.1288994)
Supplement: Supplementary file 5 [file Table4.DOCX]

| **Marker** | **6-OHDA-WT Mice** | | **6-OHDA-P2X4KO Mice** | | ***t* test value** | **p-value** |
| --- | --- | --- | --- | --- | --- | --- |
|  | **Mean ± SEM** | **Number of mice** | **Mean ± SEM** | **Number of mice** |  |  |
| **IL-18** | 1.05 ± 0.04 | 5 | 1.33 ± 0.03 | 7 | t=5.922; df=10 | p=0.000147 |
| **IL-16** | 0.90 ± 0.07 | 4 | 1.09 ± 0.05 | 8 | t=2.343; df=10 | p=0.041145 |
| **TNF-α** | 0.44 ± 0.10 | 4 | 1.36 ± 0.11 | 8 | t=5.340; df=10 | p=0.000328 |
| **TNF-R** | 0.97 ± 0.06 | 5 | 1.53 ± 0.09 | 8 | t=4.755; df=11 | p=0.000594 |
| **IL-6** | 1.29 ± 0.05 | 4 | 0.85 ± 0.12 | 7 | t=2.571; df=9 | p=0.030130 |
| **CCR5** | 0.91 ± 0.05 | 5 | 1.44 ± 0.04 | 8 | t=7.986; df=11 | p=0.000007 |
| **CX3CL1** | 0.95 ± 0.04 | 5 | 1.42 ± 0.04 | 8 | t=7.04; df=11 | p=0.000021 |
| **CX3CR1** | 0.92 ± 0.04 | 5 | 1.36 ± 0.04 | 8 | t=8.132; df=11 | p=0.000006 |
| **CXCL12** | 0.81 ± 0.10 | 5 | 1.13 ± 0.04 | 8 | t=3,389; df=11 | p=0.006043 |
| **NLRP3** | 0.67 ± 0.05 | 4 | 0.92 ± 0.08 | 7 | t=2.278; df=9 | p=0.048738 |
| **IRF5** | 0.95 ± 0.08 | 5 | 1.37 ± 0.08 | 8 | t=3.783; df=11 | p=0.003030 |
| **IRF8** | 0.91 ± 0.01 | 4 | 1.38 ± 0.07 | 8 | t=4.393; df=10 | p=0.001349 |
| **GSK3β** | 1.08 ± 0.06 | 5 | 1.29 ± 0.02 | 7 | t=4.051; df=10 | p=0.002319 |
| **NF-κB** | 1.02 ± 0.05 | 5 | 1.31 ± 0.04 | 8 | t=4.441; df=11 | p=0.000993 |
| **Cathepsin S** | 1.01 ± 0.04 | 5 | 1.39 ± 0.06 | 8 | t=4.750; df=11 | p=0.000600 |
| **GFAP** | 1.31 ± 0.06 | 5 | 2.17 ± 0.14 | 8 | t=4.679; df=11 | p=0.000672 |
| **SOD1** | 1.16 ± 0.06 | 5 | 1.54 ± 0.07 | 8 | t=3.657; df=11 | p=0.003776 |
| **TGF-β** | 0.93 ± 0.06 | 4 | 1.12 ± 0.02 | 8 | t=3.570; df=10 | p=0.005099 |
| **Arg1** | 0.89 ± 0.02 | 3 | 1.14 ± 0.06 | 8 | t=2.510; df=9 | p=0.033322 |

**Table S4**
